# Draft Genome of the Sea Cucumber *Holothuria glaberrima*, a Model for the Study of Regeneration

**DOI:** 10.3389/fmars.2021.603410

**Published:** 2021-04-15

**Authors:** Joshua G. Medina-Feliciano, Stacy Pirro, Jose E. García-Arrarás, Vladimir Mashanov, Joseph F. Ryan

**Affiliations:** 1 Biology Department, University of Puerto Rico, Río Piedras Campus, San Juan, PR, United States; 2 Iridian Genomes, Inc., Bethesda, MD, United States; 3 Wake Forest Institute for Regenerative Medicine, Winston Salem, NC, United States; 4 Whitney Laboratory for Marine Bioscience, University of Florida, St. Augustine, FL, United States

**Keywords:** holothuroid, echinoderm, regeneration, melanotransferrin, mitochondrial genome

## Abstract

Regeneration is one of the most fascinating and yet least understood biological processes. Echinoderms, one of the closest related invertebrate groups to humans, can contribute to our understanding of the genetic basis of regenerative processes. Among echinoderms, sea cucumbers have the ability to grow back most of their body parts following injury, including the intestine and nervous tissue. The cellular and molecular events underlying these abilities in sea cucumbers have been most extensively studied in the species *Holothuria glaberrima*. However, research into the regenerative abilities of this species has been impeded due to the lack of adequate genomic resources. Here, we report the first draft genome assembly of *H. glaberrima* and demonstrate its value for future genetic studies. Using only short sequencing reads, we assembled the genome into 89,105 scaffolds totaling 1.1 gigabases with an N50 of 25 kilobases. Our BUSCO assessment of the genome resulted in 894 (91.4%) complete and partial genes from 978 genes queried. We incorporated transcriptomic data from several different life history stages to annotate 51,415 genes in our final assembly. To demonstrate the usefulness of the genome, we fully annotated the melanotransferrin (*Mtf)* gene family, which have a potential role in the regeneration of the sea cucumber intestine. Using these same data, we extracted the mitochondrial genome, which showed high conservation to that of other holothuroids. Thus, these data will be a critical resource for ongoing studies of regeneration and other studies in sea cucumbers.

## INTRODUCTION

Regeneration, the replacement of lost or damaged body parts, is one of the most fascinating biological processes. Regenerative abilities are widespread in the animal kingdom (Bely and Nyberg, 2010), but our understanding of the evolution of regenerative capacity is based primarily on a small number of animal species. As one of the closest invertebrate relatives to humans, echinoderms represent an important model for understanding the origin and evolution of vertebrates, as well as being a model for understanding human biology. Echinoderms, along with hemichordates make up the clade Ambulacraria, the sister lineage to chordates (Cannon et al., 2016). Unlike chordates, many echinoderms, especially sea cucumbers, have extensive regenerative abilities. Nevertheless, relatively few genomic resources exist for sea cucumber models (Long et al., 2016; Zhang et al., 2017).

Sea cucumbers (holothurians) have the ability to regenerate their intestine following evisceration (García-Arrarás et al., 1998) and their radial nerve cord following transection (San Miguel-Ruiz et al., 2009). Many cellular events that underlie these abilities have been best described in the species *Holothuria glaberrima.* For instance, details of cell migration, dedifferentiation, proliferation and apoptosis during intestinal regeneration have been published (García-Arrarás et al., 2018). While we know exactly where and when these events occur at the cellular level, the underlying molecular infrastructure has not yet been clearly elucidated (Mashanov and García-Arrarás, 2011). In recent years, we have reported gene expression profiles of various stages of regeneration, providing information on which genes play important roles in sea cucumber regenerative processes (Ortiz-Pineda et al., 2009; Mashanov et al., 2014). These RNA-Seq analyses have led to interesting findings, but the lack of a reference genome has limited the impact of these results.

Here we report the nuclear and mitochondrial genomes of *H. glaberrima.* We show the utility of our draft nuclear genome by using it to ascertain the genomic structure of melanotransferrins, proteins that might play important roles in the regeneration process (Rojas-Cartagena et al., 2007; Hernández-Pasos et al., 2017). Melanotransferrins are membrane-bound molecules involved in many vertebrate biological processes and diseases, including development, cancer, and Alzheimer’s disease (Rahmanto et al., 2012). Moreover, in the sea cucumber, melanotransferrin genes are highly expressed in the intestine after immune activation (Ramírez-Gómez et al., 2009). Previously it was reported, based on transcriptomic data, that *H. glaberrima* contains four different melanotransferrin (*Mtf* ) genes (Hg*MTF1*, Hg*MTF2*, Hg*MTF3*, and Hg*MTF4*), while only one melanotransferrin is reported in all other echinoderms sampled to date (Hernández-Pasos et al., 2017). The continuous sequencing of new species has shown that certain *Teleostei* species appear to contain a single duplication of their *Mtf* gene, which is not surprising due to a whole genome duplication event that occurred in the lineage leading to the last common ancestor of extant teleosts (Jaillon et al., 2004). It has been hypothesized that the genomes from most species contain only one melanotransferrin because multiple copies may act as a dominant lethal (Lambert et al., 2005). The four melanotransferrin genes found in *H. glaberrima* are highly expressed at different stages of intestinal regeneration (Hernández-Pasos et al., 2017). For these reasons, analyzing the genomic characteristics and evolutionary history of these four melanotransferrin genes will shed light on this gene family and will facilitate further studies on their specific role and regulation.

Comparisons of mitochondrial genomes, the remnants of an ancient endosymbiotic event that led to the origin of eukaryotes (Martin et al., 2015), provide insight into the evolution of aerobic respiration across the tree of life. As part of our sequencing, assembly, and annotation of the nuclear genome, we have also done the same for the mitochondrial genome of *H. glaberrima*. In addition, we have compared the *H. glaberrima* mitochondrial genome with two other published holothuroid mitochondrial genomes.

The nuclear genome of *H. glaberrima* will be valuable resource for ongoing investigations into regenerative processes in this animal. In addition, both nuclear and mitochondrial genomic data will be useful for future studies in systematics, immunology, and evolution, among others.

## MATERIALS AND METHODS

### Data Collection

**FIGURE 1 |:**
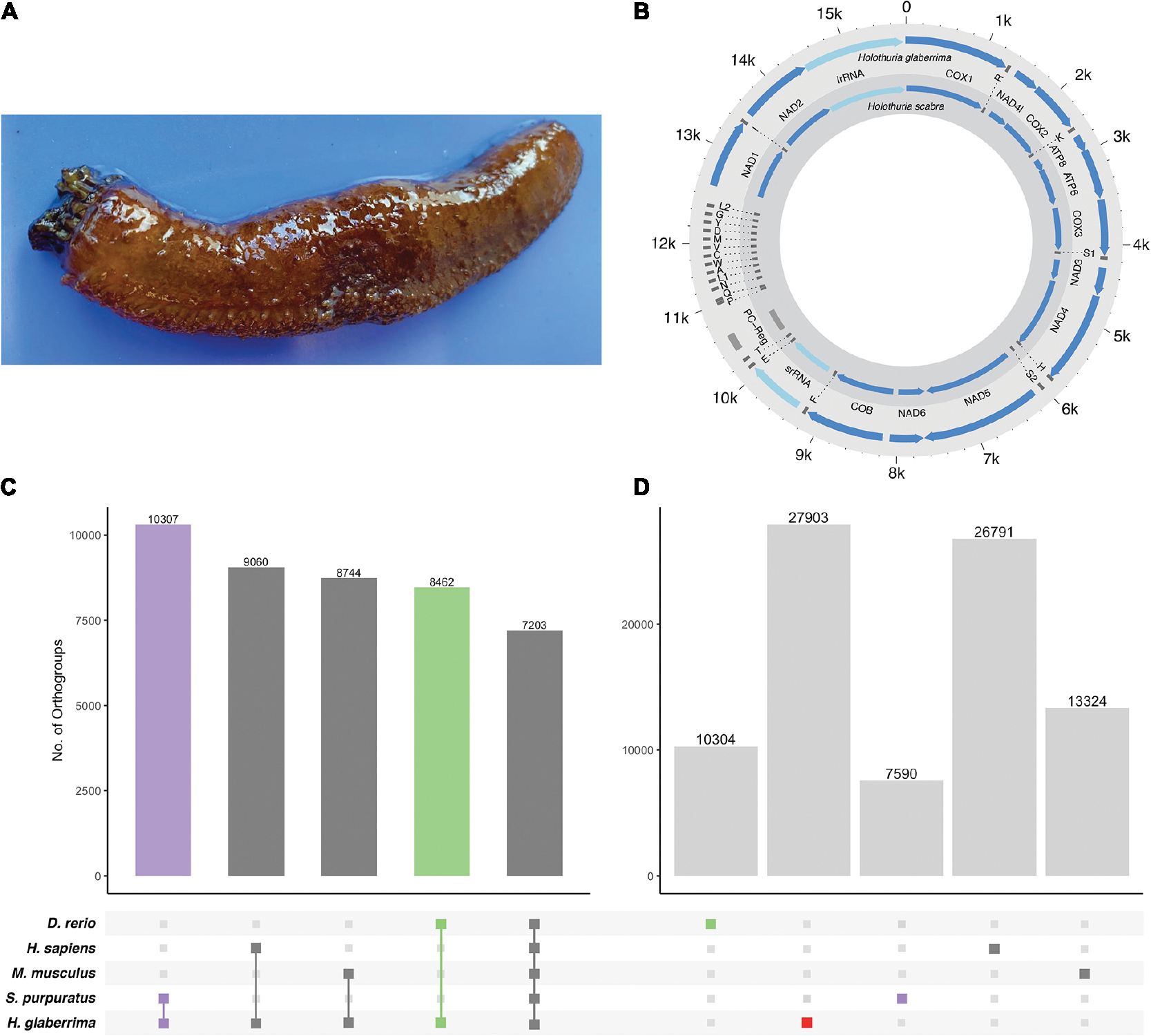
*H. glaberrima* mitochondrial genome and orthogroup analysis. **(A)** The sea cucumber *H. glaberrima*. **(B)** Comparison of mitochondrial features of *H. glaberrima* (outer) and *H. scabra* (inner). Genes = blue, rRNA = light blue, tRNA = dark gray, PC region = light gray. **(C,D)** Gene content distribution in echinoderm and vertebrate lineages. Filled circles in the bottom panel indicate **(C)** shared and **(D)** species-specific orthogroups in those lineages. Bar graphs indicate the number of orthogroups corresponding to each filled-circle pattern. **(C)** OrthoFinder analyses were performed with amino acid sequences of *H. glaberrima* protein models (based on predicted gene models), *D. rerio, H. sapiens, M. musculus*, and *S. purpuratus*. Total orthogroup overlap between *H. glaberrima* and each other species as well as total orthogroups shared between all species. **(D)** Species-specific orthogroups (OGs) for all species analyzed. These include singlets – sequences that didn’t form a group with any other sequence in the analysis and orthogroups consisting of only sequences from a single species.

A single adult male sea cucumber (Figure 1A) was collected from northeastern Puerto Rico and kept in aerated sea water until dissection. Gonads were collected into ethanol and sent to Iridian Genomes Inc. for DNA extraction, library preparation, and sequencing (Bethesda, MD). Library was prepared using the Illumina TruSeq kit with recommended 600 bp insert size following manufacturer’s standard procedure.

### Pre-assembly Processing

Sequencing was performed using an Illumina HiSeq X Ten instrument, which generated a total of 421,770,588 paired-end reads (126.5 gigabases) of 150 nucleotides each. These reads were deposited in NCBI’s Short Read Archive (SRA) (Leinonen et al., 2011) under the accession SRR9695030 within 1 week of sequencing. Adapters from the raw sequencing reads were trimmed using Trimmomatic v0.39 (ILLUMINACLIP:2:30:10 LEADING:3 TRAILING:3 SLIDING-WINDOW:4:15 MINLEN:36) (Bolger et al., 2014). We used ALLPATHS-LG v.44837 ErrorCorrectReads.pl script (Gnerre et al., 2011) to apply error correction to reads that had been trimmed for adapters and genome size estimation.

We next created filtered mitochondrial reads from the resulting trimmed/error-corrected reads. To do this we generated a preliminary assembly using Platanus Genome Assembler v1.2.4 (Kajitani et al., 2014) with *k* = 45 and then performed a BLASTN search against this assembly using the *Holothuria scabra* mitochondrial genome as a query. We identified a single contig that represented the entire mitochondrial genome. We then used FastqSifter v1.1.3 (Ryan, 2015a) to remove all trimmed/error-corrected reads that aligned to the *H. glaberrima* mitochondrial genome. FastqSifter removed 53,304 read pairs and 3,583 unpaired reads, leaving 386,920,975 read pairs and 33,176,161 unpaired reads, which were used to assemble the final nuclear genome.

### Genome Assemblies and Annotation

We used Platanus Genome Assembler v1.2.4 using a range of kmer values (31, 45, 59, 73, and 87). From these assemblies we selected our optimal assembly (K31) based on the general statistics results (Supplementary Table 1). In order to incorporate contiguous regions brought together in suboptimal assemblies (i.e., K45, K59, K73, and K87) and absent from our K31 optimal assembly, we used Matemaker v1.0.0 (Ryan, 2015b) to generate artificial mate-pairs from our suboptimal assemblies using a range of insert sizes (Matemaker was previously used in Long et al., 2016; DeBiasse et al., 2020; Ketchum et al., 2020). In total, Matemaker produced ~160 million artificial mate pairs from the suboptimal assemblies of which, insert sizes of 2 kb made up 42%, 5 kb made up 31%, 10 kb made up 19%, and 20 kb made up 8% (Supplementary Table 2). We then used SSPACE v3.0 (Boetzer et al., 2011) using default parameters to scaffold the optimal K31 contig assembly using the generated artificial mate-pairs. Finally, we applied Redundans v0.14a (Pryszcz and Gabaldón, 2016) with default parameters to eliminate redundancy and potentially generate additional scaffolding and gap closing.

We used Braker v2.1.5 (Stanke et al., 2006, 2008; Hoff et al., 2016, 2019; Brùna et al., 2020a) to predict gene models using GeneMark v4.62 (Lomsadze et al., 2005; Brùna et al., 2020b) and Augustus v3.3.3 (Stanke and Waack, 2003). As part of this process, we generated a sorted bam file from RNA sequencing data of non-regenerative and 1 and 3 days regenerative intestinal tissue (NCBI BioProject: PRJNA660762) by aligning unassembled reads to our genome assembly using STAR v.2.7.0e (Dobin et al., 2013). We also utilized protein sequences of *Strongylocentrotus purpuratus* (Spur_3.1) downloaded from ENSMBL to generate protein hints with ProtHint (Iwata and Gotoh, 2012; Gotoh et al., 2014; Buchfink et al., 2015; Brùna et al., 2020b). Both the RNA-Seq alignments and hints were utilized by Braker for its hybrid annotation pipeline. To test whether there was evidence for non-animal contamination in the resulting gene predictions, we screened predicted protein models using Alien_Index v2.1 (Ryan, 2014) using default parameters and the version 0.02 of the alien_index database. We identified 153 protein models with BLASTP hits to non-animal sequences with lower E-values than to hits to animal sequences. Of these, only 12 had alien index scores >45, a value deemed by Gladyshev et al. (2008) to be a good indicator of foreign origin. This result indicates that if there is non-animal contamination, it is minimal (it could be that these 12 are horizontally transferred genes). We assessed the assemblies and gene prediction models with BUSCO v3 (Simão et al., 2015) against the metazoan database of 978 genes using the web assessment tool gVolante (Nishimura et al., 2017).

### Mitochondrial Genome

While assembling the *H. glaberrima* nuclear genome, we identified the assembled mitochondrial genome. There was a 32-nucleotide overlap between the beginning and the end of the mitochondrial scaffold as would be expected from a circular genome. As such, we removed the overlap and reordered the scaffold so that the beginning of the scaffold corresponded with the beginning of the *cox1* gene. We used Mitos (Bernt et al., 2013) to predict genes and tRNAscan-SE 2.0 (Chan and Lowe, 2019) to annotate tRNAs. Every annotated gene was additionally confirmed with BLAST against *H. scabra* mitochondrial genes. This approach resulted in more precise annotation as some of the stop codons were wrongly predicted by Mitos. Furthermore, tRNAscan-SE failed to predict some of the tRNAs in the *H. glaberrima* mitochondrial genome, in which case we used Mitos predictions.

### Comparative Genomics

We used OrthoFinder v2.2.7 (Emms and Kelly, 2019) with default parameters to generate orthogroups using protein models from the newly assembled *H. glaberrima* genome, as well as from *Homo sapiens, Mus musculus, Danio rerio*, and *S. purpuratus.* The datasets from the included species for this analysis were obtained from the Ensembl FTP database.

### Melanotransferrin

**FIGURE 2 |:**
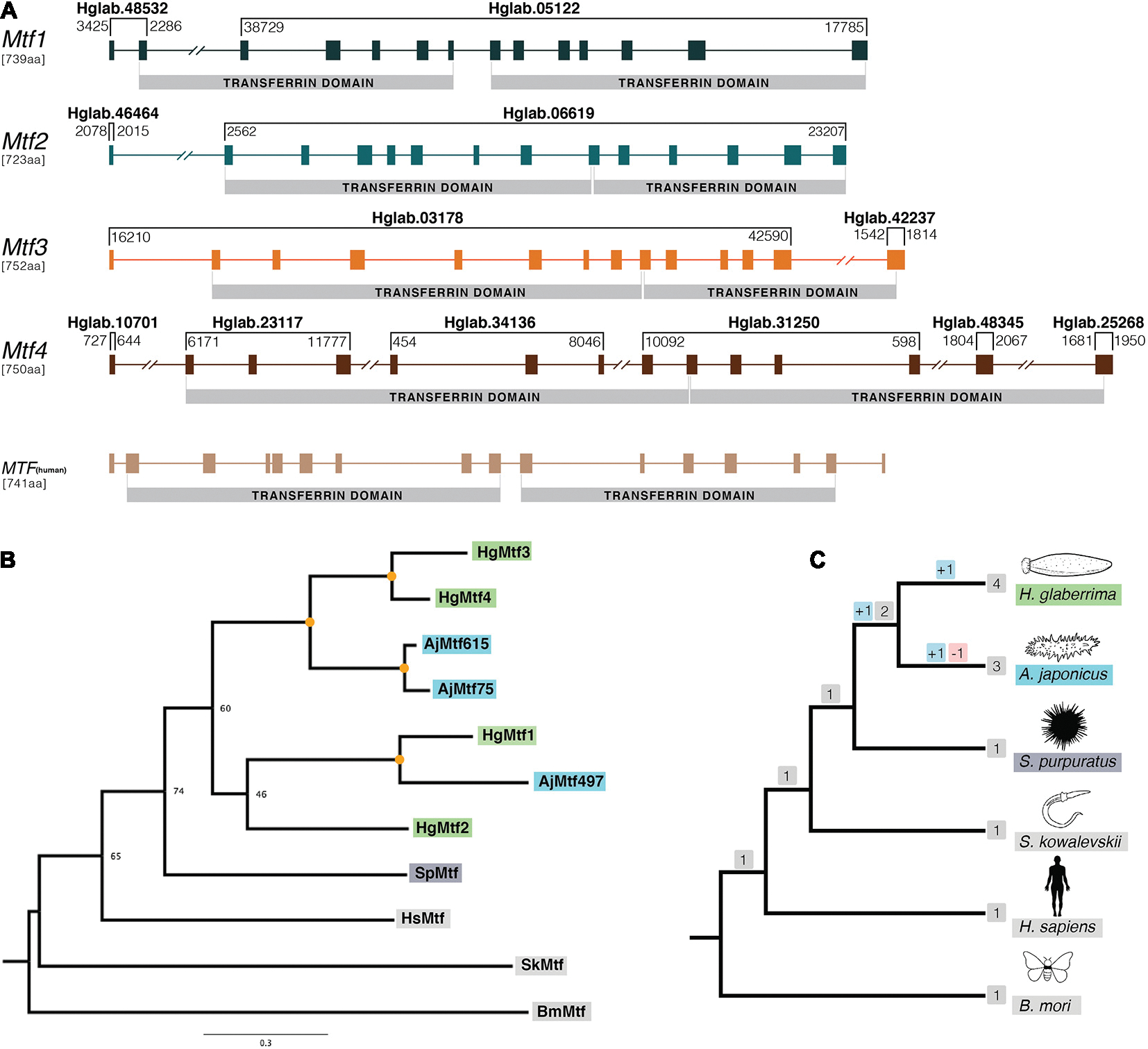
Gene structure and phylogenetic analysis of *H. glaberrima* melanotransferrin (*Mtf*) genes. **(A)** Genomic structure was determined using *H. glaberrima Mtf* gene and protein sequences present in NCBI from previous studies from Hernández-Pasos et al. (2017). Scaffolds containing specific exons of the genes are shown at the top of each bracket (e.g., Hglab.48532). Coordinates of the sequence in each scaffold are shown among brackets based on genome nucleotide sequence. Human *Mtf* gene structure was acquired from NCBI genome browser (*MELTF* NCBI: NM_005929.6). Transferrin domains are shown at the bottom of each gene. **(B)** Gene tree of all *Mtf* genes from *H. glaberrima (Hg)*, *A. japonicus (Aj), S. purpuratus (Sp), H. sapiens (Hs), S. kowalevskii (Sk),* and *B. mori (Bm). Mtf* gene sequences of the last three species were acquired from NCBI. *A. japonicus Mtf* gene names represent the scaffold number where they are found in its genome assembly. Orange circles in nodes indicate bootstrap values of 100. **(C)** Species tree of organisms used in the phylogenetic analysis of the *Mtf* genes. Gene duplication and loss are shown in red and blue squares, respectively.

We further evaluated our genome assembly by closely examining *H. glaberrima* genes that had been previously described in studies based on expressed sequence tags (EST), RNA sequencing, and/or Sanger-based cDNA sequencing. This approach allowed us to manually assess the accuracy of our genome assembly and to determine gene structures. Specifically, we selected the melanotransferrin gene family which is significantly upregulated in *H. glaberrima* during intestine regeneration (Rojas-Cartagena et al., 2007). This group included *HgMtf1* (NCBI: GQ243222), *HgMtf2* (NCBI: KP861416), *HgMtf3* (NCBID: KP861417), and *HgMtf4* (NCBI: KP861418). We aligned four previously identified Mtf genes to our genome assembly using NCBI BLASTN (Altschul et al., 1990) (Figure 2A and Supplementary Figures 1, 2). Based on these alignments and 5′-GT and AG-3′ intron boundaries, we manually annotated the four Mtf genes across multiple scaffolds. The presence of these Mtf genes was further confirmed by BLAST searches against a previously unpublished transcriptome assembly^1^. Multiple sequence alignments were done using MAFFT v7.471 (Katoh and Standley, 2013).

To deduce the relationship between the four paralogous *H. glaberrima Mtf* genes with those of other animals, we conducted a phylogenetic analysis (Figures 2B,C). We used *Mtf* genes from *H. glaberrima*, *Apostichopus japonicus, S. purpuratus, Saccoglossus kowalevskii, H. sapiens,* and *Bombyx mori.* We also performed a phylogenetic analysis including *Parus major* (bird), *Ciona intestinalis* (tunicate), *Etheostoma cragini* (teleostei), and *Exaiptasia diaphana* (cnidaria) (Supplementary Figure 3). For both phylogenetic trees performed, we used a comparative approach using IQ-TREE v2.03 and RAxML v8.2.12 (Stamatakis, 2014; Nguyen et al., 2015). The phylotocol (DeBiasse and Ryan, 2019) laying out the planned phylogenetic analyses *a priori* can be found in the article’s GitHub repository (josephryan/Holothuria_glaberrima_draft_genome).

## RESULTS AND DISCUSSION

### Genome Assembly and Annotation

After removing adapter sequence with Trimmomatic, 400,075,279 (95%) read pairs were retained and 4,200,427,854 (3.5%) bases from these retained pairs were trimmed. After error correction of reads with the ErrorCorrectReads.pl script from ALLPATHS-LG we removed a total of 1,104,390 read pairs (0.1%), orphaned (removed 1 from a pair) an additional 12,545,131 read pairs and made 117,247,709 corrections to 95,509,388 (11.6%) reads (an average of 1.2 corrections per corrected read). The genome size estimation generated by ErrorCorrectReads.pl was 1.2 gigabases (1,171,092,004 bases) with a coverage estimate of 84×. This number is close to the estimate obtained with Feulgen densitometry (~1.4 gigabases) (Supplementary Figure 4).

We generated five assemblies using Platanus with a range of kmer parameters (31, 45, 59, 73, and 87). The assembly with the kmer parameter K = 31 resulted in the highest N50 (11,332) (Supplementary Table 1). After scaffolding the optimal K31 assembly with artificial mate-pairs from the suboptimal assemblies (K45, K59, K73, and K87), we produced an assembly consisting of 2,960,762 scaffolds comprising 1,468,331,553 bases with an N50 of 15,069 bases. We next ran the program Redundans to reduce the overall redundancy due to heterozygosity in the data. After Redundans, our assembly consisted of 89,105 scaffolds comprising 1,074,346,208 bases (close to the size estimations from both kmers and Feulgen densitometry) and an N50 of 25,282 bases. The longest scaffold was 244,429 nucleotides. After applying Redundans, there was an improvement in the percentage of scaffolds greater than 50 kb from 11.9 to 18.7%. BUSCO assessment of our final assembly resulted in recovering 750 (76.7%) complete and 144 (14.7%) partial genes for a total of 894 (91.4%) found genes from the 978 queried genes in the metazoa dataset (Supplementary Table 3). Comparative statistics and BUSCO assessments for assemblies before and after applying Redundans can be found in Supplementary Table 3.

We predicted 53,080 gene models with a mean exon length of 185 bp and mean intron length of 1916 bp as calculated using GenomeFeatures v3.10 (Lawrence et al., 2013). Of these 53,080 gene models, 1,665 represented additional isoforms, and the total number of uniquely predicted genes was 51,415. We estimate that more than 40% of the *H. glaberrima* genes are represented by more than one gene model and infer that this is likely due to the fractured nature of the genome. We therefore suspect the number of genes is likely substantially lower than the number of gene models (Supplementary Figure 5). Our BUSCO assessment of translated gene models resulted in the recovery of 798 (81.6%) complete and 164 (16.8%) partial genes for a total of 962 (98.4%) from the 978 queried genes in the Metazoa dataset (Supplementary Table 4). We also performed gene predictions on our initial assembly, after manually eliminating scaffolds shorter than 200 bp, to make comparisons to that of our predictions using our final assembly after Redundans. To validate our removal of redundant scaffolds, we annotated the assembly before and after applying Redundans and assessed these annotations with BUSCO. The BUSCO assessment of the 58,944 protein models produced from the assembly prior to running Redundans resulted in 778 (79.6%) complete and 181 (18.5%) partial genes, which is substantially inferior to our final annotation (Supplementary Table 4).

### Mitochondrial Genome

The *H. glaberrima* mitochondrial genome is 15,617 bp, similar in size to that of previously reported holothuroids (*H. scabra* = 15,779 bp, *H. leucospilata* = 15,904 bp) (Perseke et al., 2010). Likewise, mitochondrial genome features of *H. glaberrima* showed the same gene order reported for *H. scabra* and similar characteristics to those of *H. scabra* and *H. leucospilata* (Figure 1B and Supplementary Table 5) (Xia et al., 2016; Yang et al., 2019). Differences included the length of the putative control region (PC region) which was smaller in *H. glaberrima* with 272 bp compared to 456 bp and 551 bp for *H. scabra* and *H. leucospilata*, respectively. Moreover, the total number of intergenic nucleotides in *H. glaberrima* (113 bp) was similar to *H. scabra* (109 bp). Total intergenic nucleotides were distributed among 15 intergenic spacers, ranging from 1 to 20 bp in size, comparable to *H. scabra* and *H. leucospilata* which contain 15 and 16 intergenic spacers, respectively. There were 5 regions with overlapping genes within the genome, the most significant being those between *atp8* and *atp6*, and *nad4* and tRNA-His, with overlaps of 7 bp, and tRNA-P and tRNA-Q with overlap of 4 bp. From the 5 overlapping regions, *H. glaberrima* shares all the mentioned overlapping areas with *H. scabra* and *H. leucospilata* suggesting that these are ancient overlaps. In addition, as reported for both *H. scabra* and *H. forskali*, *H. glaberrima* has an incomplete stop codon T of *cox2*, supporting the likeliness of it being a distinctive feature of Holothuria (Perseke et al., 2010; Xia et al., 2016).

### Comparative Genomics

Our OrthoFinder analysis resulted in a total of 23,126 orthogroups. We identified 7,203 orthogroups shared between all species, 10,307 that contained only sequences from *H. glaberrima* and S. *purpuratus*, and close to 9,000 for each of the groups that included only *H. glaberrima* and one of the vertebrates (Figure 1C). The high number of orthogroups shared between *S. purpuratus* and *H. glaberrima* highlights the quality of the predicted gene models. We identified 27,903 *H. glaberrima*-specific orthogroups; this included 107 that consisted of multiple *H. glaberrima* protein models per orthogroup and 27,796 consisting of only a single *H. glaberrima* protein model (“singlets”). There were far more species-specific orthogroups in *H. glaberrima* than there were in any of the other species—*H. sapiens* was next with 26,791, which we attribute to the high amount of peptide sequences in the dataset (102,915) (Figure 1D).

### Melanotransferrin

The human *Mtf* gene (*MELTF*) is large, coding for 749 amino acids and its 16 exons span more than 28 kb. The *H. glaberrima Mtf* genes are similarly large (Hg*Mtf1* = 739 aa, Hg*Mtf2* = 723 aa, Hg*Mtf3* = 752 aa, Hg*Mtf4* = 750 aa), with all four genomic loci having 14 exons and spanning many kilobases. Due to the large size of these genes and the fragmented nature of our assembly, all four of the *Mtf* genes occur on multiple scaffolds (Figure 2A). Nevertheless, we were able to account for all exons and capture each gene structure. Besides differing from the human *Mtf* gene in the number of exons, variations occur in the position of several introns suggesting that there have been substantial structural changes in these genes since the last common ancestor of vertebrates and echinoderms.

Our assembly captures considerable variability between the Hg*Mtf1* and the three other *H. glaberrima Mtf* genes. For example, the sequence that codes for the N-terminal transferrin domains of HgMtf1 is interrupted by 5 introns, whereas the corresponding coding regions in Hg*Mtf2–4* include 7 introns. Likewise, the sequence that codes for the C-terminal transferrin domain of Hg*Mtf1* includes 6 introns, whereas these same coding regions in Hg*Mtf2–4* include 5 introns (Figure2A). These results show that there have been considerable evolutionary changes in the genomic architecture Hg*Mtf1*, suggesting that *HgMtf1* did not arise recently. A sequence alignment of all the annotated *Mtf* proteins is shown in Supplementary Figure 2.

The 3′ sequence of Hg*Mtf1* in the genome differs from the previously reported cDNA sequence in GenBank (ACS74869) (Supplementary Figure 1). There appears to be a single nucleotide present in the genome that is absent from the previously reported sequence. This frameshift leads to 65 fewer amino acids in the translation of the genomic Hg*Mtf1*. We surmise that the genome is correct and that the previously published sequence includes an erroneous single-nucleotide deletion based on the following evidence: a BLASTP of the 65 amino-acid portion against the GenBank non-redundant database produces only a single match to itself. The region in question is exactly the same in all of our optimal and suboptimal assemblies. Moreover, TBLASTN of the 65 amino acids against our transcriptome produced no hits. Alignment of Hg*Mtf1* against the previous GenBank sequence and the Hg*Mtf1* recovered from our transcriptome also confirms this erroneous frameshift (Supplementary Figure 1).

The other sea cucumber species, *A. japonicus*, in our orthogroup analysis, included more than one *Mtf* gene in its genome (Zhang et al., 2017). Initial pre-genomic studies reported only one melanotransferrin in *A. japonicus* (Qiu et al., 2014), but the latest genome data showed that there were multiple copies in its genome. We searched the predicted gene models of *A. japonicus* and found three *Mtf* genes, which we included in our phylogenetic analysis (our criteria for inclusion were the presence of two full transferrin domains). The gene tree shown in Figure 2B, demonstrate the relationship between the *A. japonicus* and *H. glaberrima* Mtf genes. As such, our phylogenetic analysis suggests that some *Mtf* gene-family expansion occurred in the stem of the holothuroid lineage but that additional duplications occurred independently in the lineages leading to *A. japonicus* and *H. glaberrima* (Figures 2B,C). Recent evidence of data deposited in NCBI suggest that also certain teleost species might contain a duplicated *Mtf* gene (i.e., *E. cragini*). However, it has been predicted that one of these *E. cragini Mtf* genes is no longer functional (Glasauer and Neuhauss, 2014). Our phylogenetic results suggest that the four *A. japonicus* and four *H. glaberrima Mtf* genes are not one-to-one orthologs, which would suggest that they each inherited these from the last common ancestor of these two lineages. Instead, under parsimony, our results suggest the following: this last common ancestor had at least 3 *Mtf* genes (i.e., MTF1, MTF2, and MTF3/4), MTF2 was lost in *A. japonicus*, MTF1 and MTF3/4 duplicated in *A. japonicus*, and MFTF3/4 duplicated in *H. glaberrima*.

## CONCLUSION

We have sequenced, assembled, and annotated the first draft genome of *H. glaberrima*, one of the best studied regenerative model invertebrates. We demonstrated the potential of the assembled genome by analyzing the *Mtf* gene family genomic loci. We also assembled and annotated the *H. glaberrima* mitochondrial genome showing conservation with other mitochondrial genomes from the genus *Holothuria*. Our work provides a key resource for future studies in animal regeneration and beyond.

## Supplementary Material

Table_1

Table_2

Table_3

Table_4

Data_Sheet_1

Table_5

## Data Availability

The datasets presented in this study can be found in online repositories. The names of the repository/repositories and accession number(s) are as follows: the raw genomic reads are available at NCBI’s SRA (Accession: SRR9695030). The genome assembly is available at http://ryanlab.whitney.ufl.edu/genomes/Holothuria_glaberrima/ and at NCBI (PRJNA497079). The RNA sequencing reads used for the annotation are available in NCBI under the BioProject PRJNA660762. Custom scripts, command lines, and data used in these analyses and alignment and tree files are available at https://github.com/josephryan/Holothuria_glaberrima_draft_genome.

## Footnotes

1
http://ryanlab.whitney.ufl.edu/genomes/Holothuria_glaberrima/
