# Supplementary material for: Draft Genome of the Sea Cucumber Holothuria glaberrima, a Model for the Study of Regeneration
Source: Front Mar Sci. Author manuscript; Available in PMC 2024 May 13. (PMC11090492; doi:10.3389/fmars.2021.603410)
Supplement: Table_1 [file NIHMS1988039-supplement-Table_1.docx]

| **Table S1.** General Statistics of Suboptimal Assemblies from Platanus | | | |
| --- | --- | --- | --- |
| **Kmer Value** | **N50** | **# of Bases** | **# of Sequences** |
| 31 | 6931 | 1,553,348,184 | 3,781,323 |
| 43 | 6372 | 1,575,753,783 | 4,167,191 |
| 59 | 6702 | 1,493,359,613 | 3,499,740 |
| 73 | 6730 | 1,455,653,386 | 3,248,885 |
| 87 | 6298 | 1,433,361,230 | 3,088,676 |
