# Supplementary material for: Draft Genome of the Sea Cucumber Holothuria glaberrima, a Model for the Study of Regeneration
Source: Front Mar Sci. Author manuscript; Available in PMC 2024 May 13. (PMC11090492; doi:10.3389/fmars.2021.603410)
Supplement: Table_2 [file NIHMS1988039-supplement-Table_2.docx]

| **Table S2.** Artificial mate pairs produced by MateMaker from sub-optimal assemblies | | | | | |
| --- | --- | --- | --- | --- | --- |
| **Insert Size** | **K=45** | **K=59** | **K=73** | **K=97** | **Total** |
| 2 Kb | 16901924 | 16618223 | 16303024 | 16150912 | **65974083** |
| 5 Kb | 12614392 | 12262738 | 11873432 | 11670356 | **48420918** |
| 10 Kb | 8086142 | 7723665 | 7328113 | 7112040 | **30249960** |
| 20 Kb | 3573305 | 3299169 | 3035314 | 2868968 | **12776756** |
| **Total** |  |  |  |  | **157421717** |
