# Supplementary material for: Draft Genome of the Sea Cucumber Holothuria glaberrima, a Model for the Study of Regeneration
Source: Front Mar Sci. Author manuscript; Available in PMC 2024 May 13. (PMC11090492; doi:10.3389/fmars.2021.603410)
Supplement: Table_3 [file NIHMS1988039-supplement-Table_3.docx]

| **Table S3.** General Genome Scaffold Assemblies Metrics and BUSCO Completeness Assessments. | | |
| --- | --- | --- |
|  | **Initial Assembly** | **Final Assembly** |
| **General Metrics** |  |  |
| Number of bases | 2,960,762 | 89,105 |
| Number of sequences | 1.5 Gb | 1.1 Gb |
| N50 | 15.07 Kb | 25.28 Kb |
| Maximum scaffold length | 244.11 Kb | 244.43 Kb |
| No. of scaffolds > 50kb | 2,543 | 2,872 |
| %Main genome in scaffolds >50 kb | 11.90% | 18.68% |
| GC content | 38.9% | 38.5% |
| % N bases | 0.49% | 0.07% |
| **BUSCO Assessment** | |  |
| Complete | 731 (74.7%) | 750 (76.7%) |
| Complete + partial | 882 (90.2%) | 894 (91.4%) |
| Complete and single copy | 74.1% | 75.9% |
| Complete and duplicated | 0.6% | 0.8% |
| Fragmented | 151 (15.4%) | 144 (14.7%) |
| Missing | 96 (9.9%) | 84 (8.6%) |
| **Note:** BUSCO assessment was performed using metazoa gene library lineage. | | |
