# Supplementary material for: Draft Genome of the Sea Cucumber Holothuria glaberrima, a Model for the Study of Regeneration
Source: Front Mar Sci. Author manuscript; Available in PMC 2024 May 13. (PMC11090492; doi:10.3389/fmars.2021.603410)
Supplement: Table_4 [file NIHMS1988039-supplement-Table_4.docx]

| **Table S4.** BRAKER Gene Model Prediction General Statistics. | | |
| --- | --- | --- |
|  | **Initial Assembly** | **Final Assembly** |
| **Metric** | **Value** | **Value** |
| Genes | 58,944 | 53,080 |
| Exons | 224,434 | 222,439 |
| Introns | 165,627 | 169,494 |
| Mean exon length | 214 bases | 210 bases |
| Mean intron length | 1,895 bases | 1,928 bases |
| **BUSCO assessment** |  |  |
| Complete | 778 (79.6%) | 798 (81.6%) |
| Complete + partial | 959 (98.1%) | 962 (98.4%) |
| Complete and single-copy | 739 (75.6%) | 755 (77.2%) |
| Complete and duplicated | 39 (4.0%) | 43 (4.4%) |
| Fragmented | 181 (18.5%) | 164 (16.8%) |
| Missing | 19 (1.9%) | 16 (1.6%) |
| Total Core Genes Assessed | 978 | 978 |
