# Supplementary material for: Draft Genome of the Sea Cucumber Holothuria glaberrima, a Model for the Study of Regeneration
Source: Front Mar Sci. Author manuscript; Available in PMC 2024 May 13. (PMC11090492; doi:10.3389/fmars.2021.603410)
Supplement: Data_Sheet_1 [file NIHMS1988039-supplement-Data_Sheet_1.pdf]

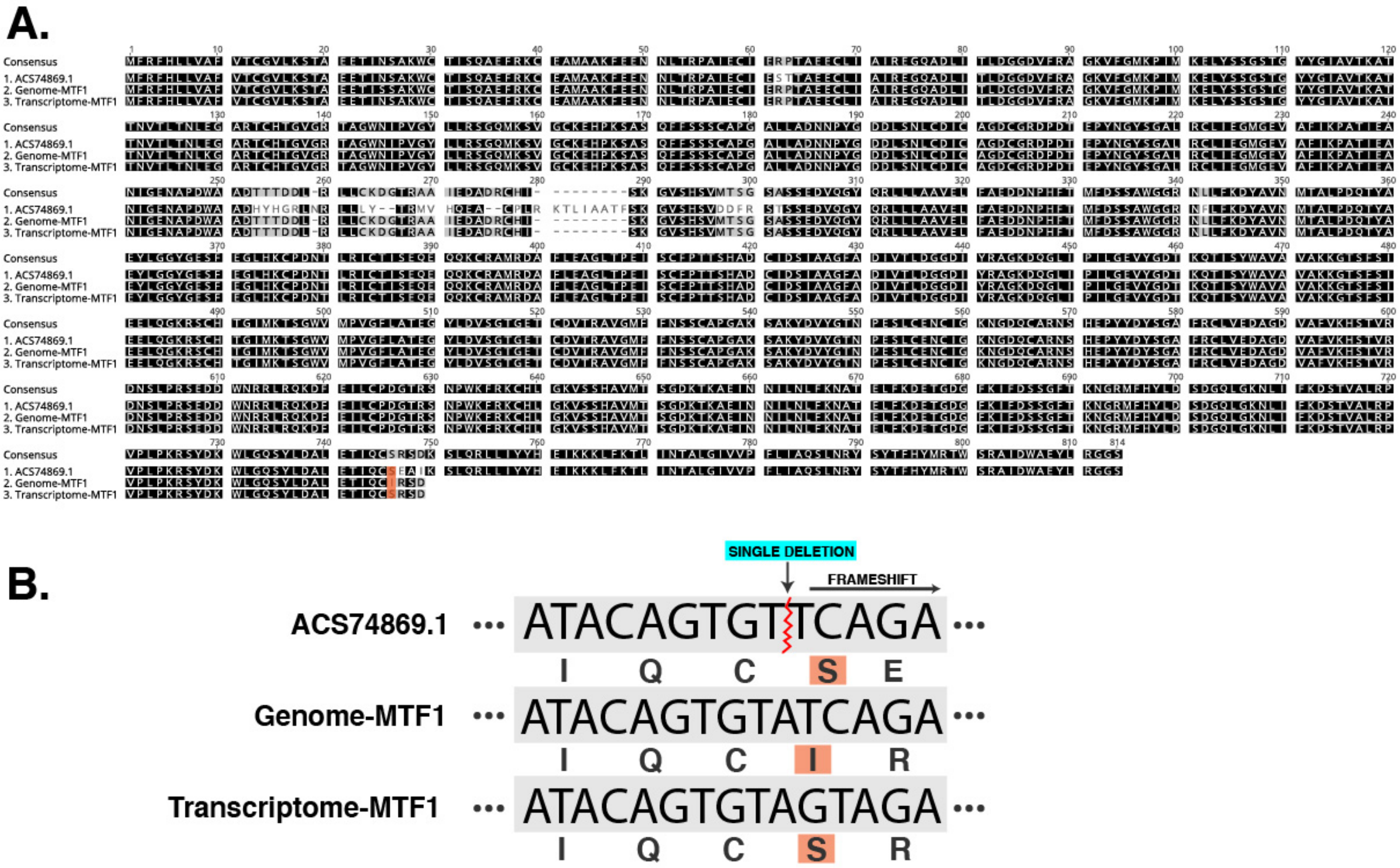

Figure S1. HgMtf1 Amino Acid Sequences Comparisons. (A) Multiple sequence alignment of *H. glaberrima* Mtf1 sequences from different sources: **1.** NCBI previously deposited sequence (ID: ACS74869.1), **2.** Genome annotated Mtf1, and **3.** Mtf1 sequence from *de novo* transcriptome data. Highlights show similarities. **(B)** Mtf1 nucleotide sequence closeup of region containing a nucleotide deletion in the previous NCBI sequence causing a frameshift. First amino acid change is highlighted in orange.

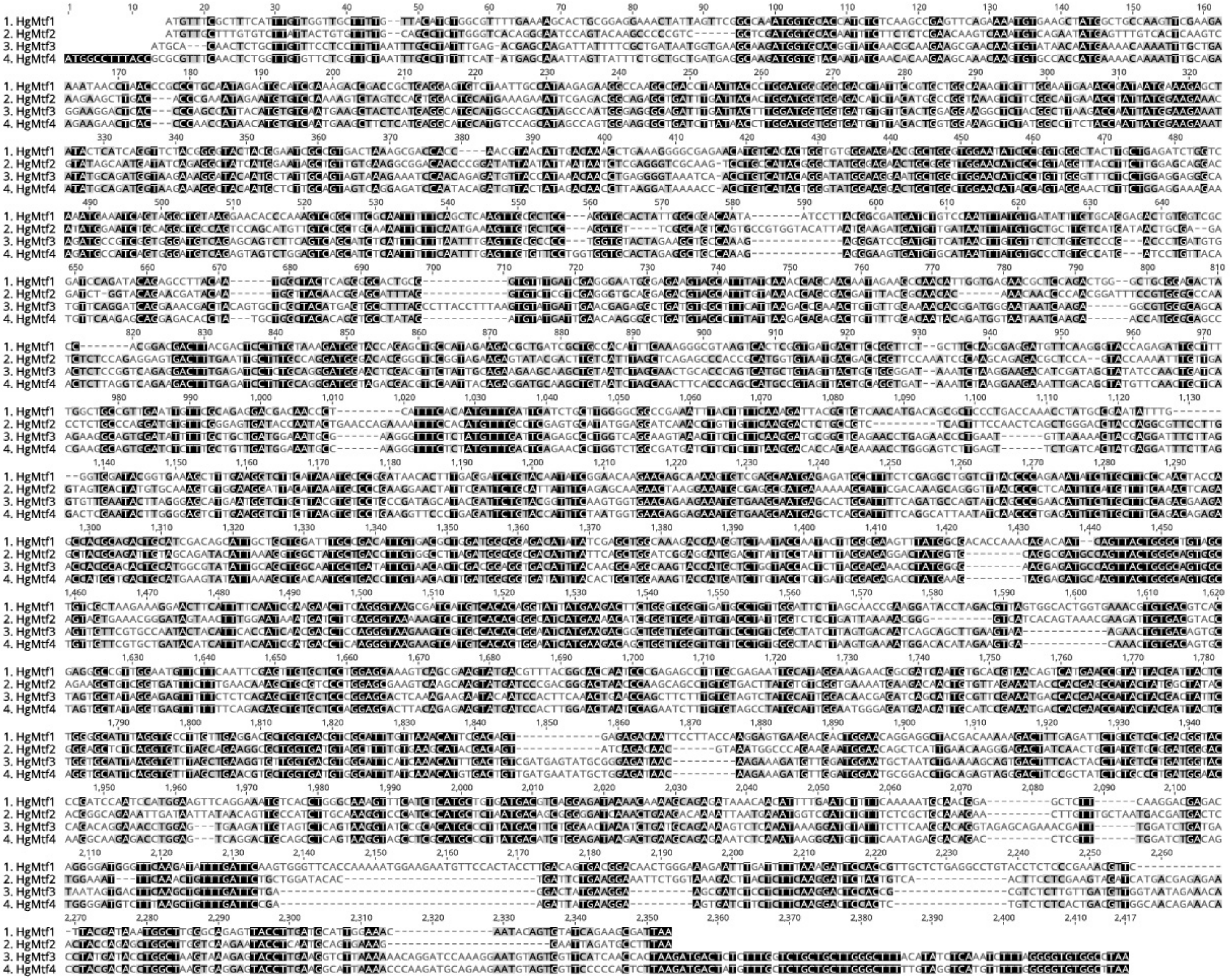

Figure S2. Multiple sequence alignment of H. glaberrima Mtf1-4 sequences annotated using our draft genome data. Highlights are based on similarity.

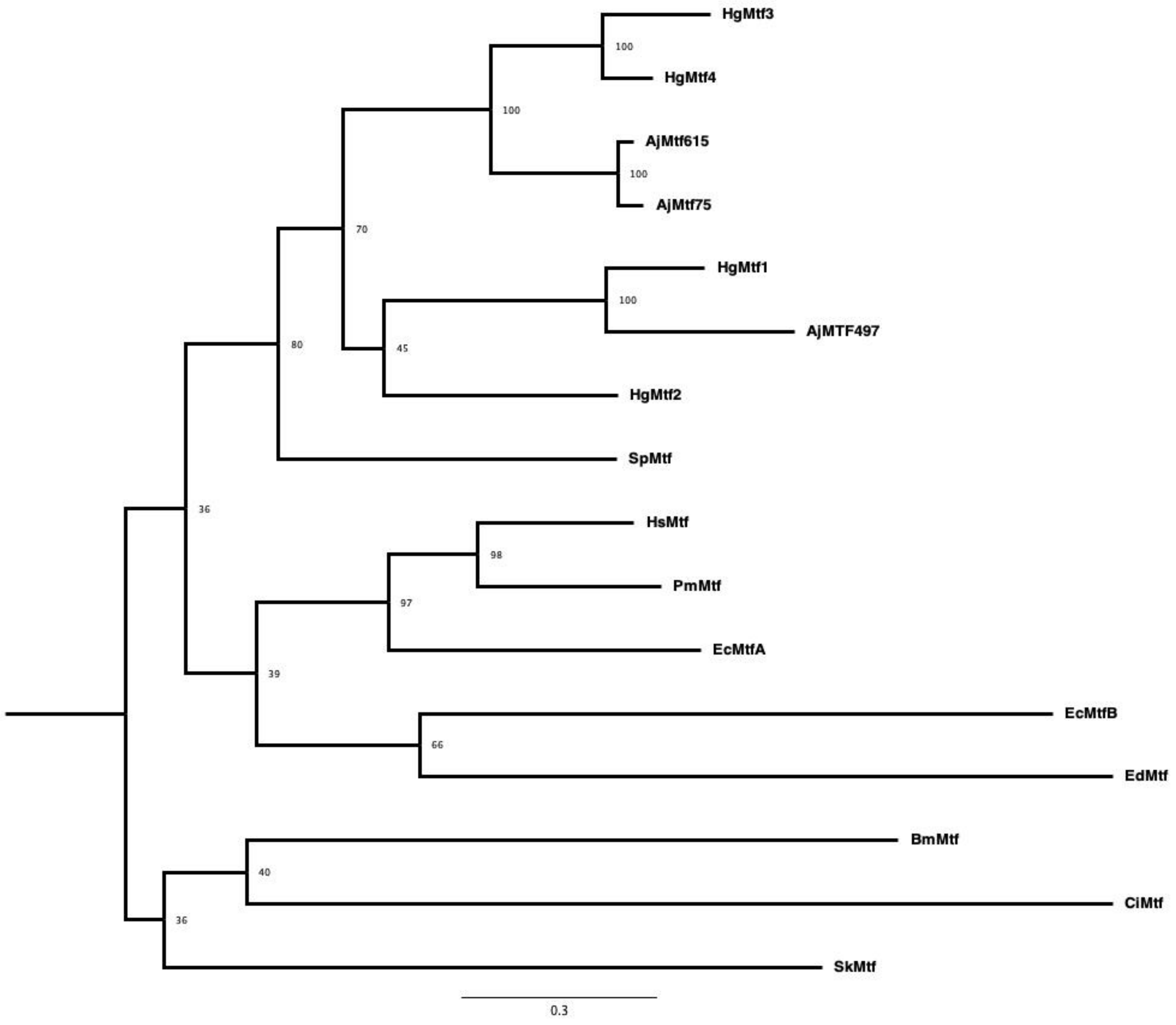

Figure S3. Broader *Mtf* gene tree containing taxa from *Holothuria glaberrima* (Hg), *Apostichopus japonicus* (Aj), *Strongylocentrotus purpuratus* (Sp), *Homo sapiens* (Hs), *S. kowalevskii* (Sk), *B. mori* (Bm), *Parus major* (Pm), *Ciona intestinalis* (Ci), *Etheostoma cragini* (Ec), and *Exaiptasia diaphana* (Ed).

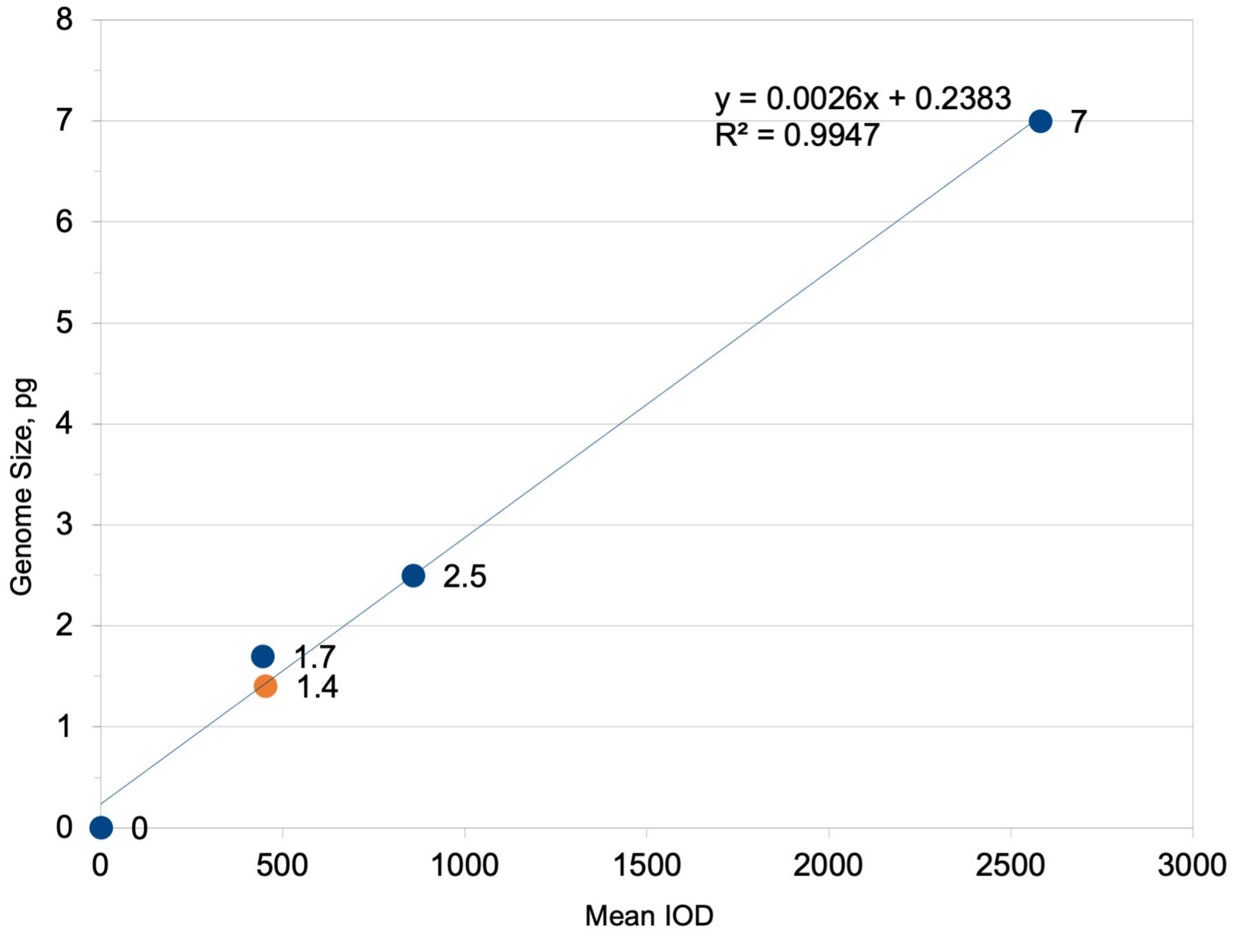

Figure S4. Feulgen densitometry analysis for *H. glaberrima* genome size estimate. Standards plot was generated using *Sclerodactyla briareus* gonads (1.7), chicken erythrocyte nuclei (2.5), and male human cheek epithelium (7). *H. glaberrima* genome size estimate was generated using gonads (orange circle -- 1.4). Total of 250 measurements were performed for *H. glaberrima*.

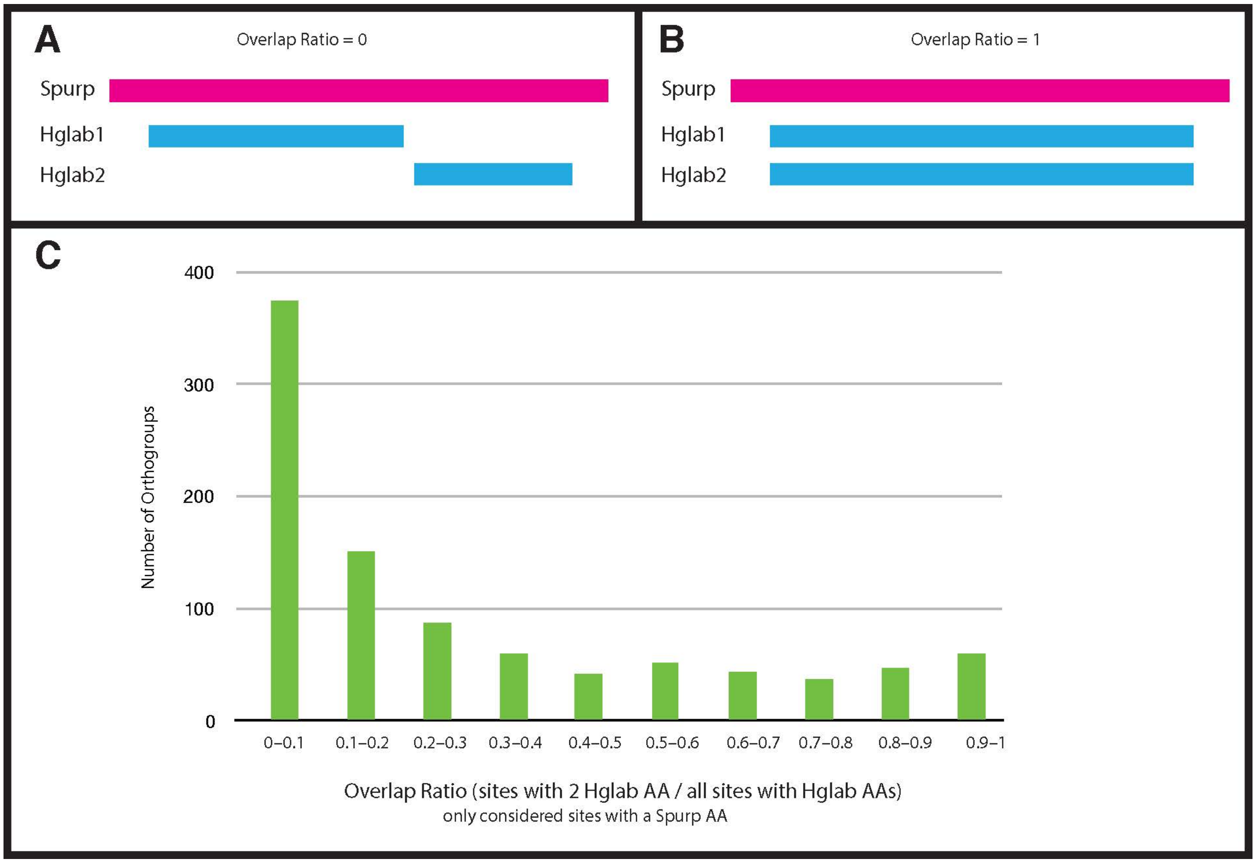

Figure S5. Evaluating gene model predictions of *H. glaberrima.* We computed overlap ratios for 954 aligned (MAFFT default parameters) orthogroups that contained one *S. purpuratus* gene model and exactly two *H. glaberrima* gene models, where the two *H. glaberrima* gene models were not isoforms of the same genomic locus. Overlap ratios were computed by dividing the number of positions in the alignment that included a non-gap character for both *H. glaberrima* sequences by the number of positions that included one or thwo non-gap characters for the *H. glaberrima sequences* (while only considering positions where the *S. purpuratus* sequence contained a non-gap character). **(A)** An example where the Overlap Ratio = 0. In this case an alignment has two non-overlapping *H. glaberrima* sequences corresponding to a single gene in *S. purpuratus.* This is likely to happen when a single gene spans two scaffolds and is represented by two gene models. **(B)** An example where the Overlap Ratio = 1. In this case, each position of the two *H. glaberrima* gene models align to each other. This is likely to happen in the case where there are two genes corresponding to a single *S. purpuratus* gene. **(C)** Almost 40% (375) of the 954 orthogroups we surveyed had an overlap ratio between 0 and 0.1, suggesting a high number of *H. glaberrima* genes being represented by more than one gene model.
