# Supplementary material for: Draft Genome of the Sea Cucumber Holothuria glaberrima, a Model for the Study of Regeneration
Source: Front Mar Sci. Author manuscript; Available in PMC 2024 May 13. (PMC11090492; doi:10.3389/fmars.2021.603410)
Supplement: Table_5 [file NIHMS1988039-supplement-Table_5.docx]

| **Table S5.** *Holothuria glaberrima* mitochondrial genome features. | | | | | | | | |
| --- | --- | --- | --- | --- | --- | --- | --- | --- |
|  |  |  |  |  | **Codon** | |  |  |
| **Gene Name** | **Strand** | **Start** | **Stop** | **Length** | **Start** | **Stop** | **Anticodon** | **Inter Nucl** |
| *cox1* | + | 1 | 1557 | 1557 | ATG | TAA |  | 11 |
| tRNA-R | + | 1568 | 1634 | 67 |  |  | TCG | 0 |
| *nad4l* | + | 1635 | 1931 | 297 | ATG | TAA |  | 0 |
| *cox2* | + | 1932 | 2619 | 688 | ATG | T-- |  | 0 |
| tRNA-K | + | 2620 | 2686 | 67 |  |  | CTT | 0 |
| *atp8* | + | 2687 | 2851 | 165 | ATG | TAA |  | -7 |
| *atp6* | + | 2845 | 3531 | 687 | ATG | TAA |  | 2 |
| *cox3* | + | 3534 | 4316 | 783 | ATG | TAA |  | -2 |
| tRNA-S^(UCA)^ | - | 4315 | 4385 | 71 |  |  | TGA | 18 |
| *nad3* | + | 4404 | 4748 | 345 | ATG | TAA |  | 0 |
| *nad4* | + | 4752 | 6116 | 1365 | ATG | TAG |  | -7 |
| tRNA-H | + | 6110 | 6179 | 70 |  |  | GTG | 1 |
| tRNA-S^(AGC)^ | + | 6181 | 6248 | 68 |  |  | GCT | 0 |
| *nad5* | + | 6249 | 8081 | 1833 | ATG | TAA |  | 17 |
| *nad6* | - | 8099 | 8587 | 489 | ATG | TAG |  | 8 |
| *cob* | + | 8596 | 9738 | 1143 | ATG | TAA |  | -1 |
| tRNA-F | + | 9738 | 9808 | 71 |  |  | GAA | 0 |
| *srRNA* | + | 9809 | 10637 | 829 |  |  |  | 1 |
| tRNA-E | + | 10639 | 10707 | 69 |  |  | TTC | 0 |
| tRNA-T | + | 10708 | 10777 | 70 |  |  | TGT | 0 |
| PC-region | + | 10778 | 11049 | 272 |  |  |  | 0 |
| tRNA-P | + | 11050 | 11116 | 67 |  |  | TGG | -4 |
| tRNA-Q | - | 11113 | 11182 | 70 |  |  | TTG | 2 |
| tRNA-N | + | 11185 | 11254 | 70 |  |  | GTT | 0 |
| tRNA-L^(CUA)^ | + | 11255 | 11326 | 72 |  |  | TAG | 5 |
| tRNA-A | - | 11332 | 11398 | 67 |  |  | TGC | 0 |
| tRNA-W | + | 11399 | 11466 | 68 |  |  | TCA | 0 |
| tRNA-C | + | 11467 | 11530 | 64 |  |  | GCA | 5 |
| tRNA-V | - | 11536 | 11605 | 70 |  |  | TAC | 20 |
| tRNA-M | + | 11626 | 11694 | 69 |  |  | CAT | 2 |
| tRNA-D | - | 11697 | 11766 | 70 |  |  | GTC | 1 |
| tRNA-Y | + | 11768 | 11835 | 68 |  |  | GTA | 0 |
| tRNA-G | + | 11836 | 11902 | 67 |  |  | TCC | 6 |
| tRNA-L^(UUA)^ | + | 11909 | 11979 | 71 |  |  | TAA | 0 |
| *nad1* | + | 11980 | 12951 | 972 | ATG | TAA |  | 14 |
| tRNA-I | + | 12966 | 13033 | 68 |  |  | GAT | 0 |
| *nad2* | + | 13034 | 14077 | 1044 | ATG | TAA |  | 0 |
| *lrRNA* | + | 14078 | 15617 | 1540 |  |  |  | 0 |
